# Electroacupuncture Stimulation at CV12 Inhibits Gastric Motility via TRPV1 Receptor

**DOI:** 10.1155/2013/294789

**Published:** 2013-09-09

**Authors:** Zhi Yu, Xin Cao, Youbing Xia, Binbin Ren, Hong Feng, Yali Wang, Jingfeng Jiang, Bin Xu

**Affiliations:** ^1^Key Laboratory of Integrated Acupuncture and Drugs Constructed by the Ministry of Education and Jiangsu Province, Nanjing University of Chinese Medicine, Nanjing 210029, China; ^2^Toho University, Tokyo 143-8540, Japan; ^3^Nanjing Medical University, Nanjing 210029, China

## Abstract

Gastric dysmotility is one of the major pathophysiological factors in functional gastrointestinal disorders. Acupuncture, as one of the alternative approaches, is efficacious in the treatment of gastrointestinal motility disorders; however, the mechanism underlying its action is unclear. In the present study, we used both capsazepine, a TRPV1 antagonist, and TRPV1 knockout mice. Animals were divided into wild-type group (WT), capsazepine injection group (CZP, 0.5 mg/kg, i.p.), and TRPV1 knockout mice group (TRPV1^−/−^). Each of these three groups was divided into three subgroups, which were subjected to EA stimulation at acupoint Zhongwan (CV12) at a different intensity (1, 2, or 4 mA). We demonstrated that electroacupuncture at Zhongwan (CV12) markedly inhibited gastric motility at 2 and 4 mA in an intensity-dependent manner in wild-type mice. The inhibitory effect was also observed in capsazepine-injected and TRPV1^−/−^ mice but was no longer intensity dependent, indicating that TRPV1 is partially involved in the electroacupuncture-mediated modulation of gastric motility.

## 1. Introduction

Gastric motility is one of the most critical physiological functions of the human body. Coordinated gastric motility is necessary for the digestion and absorption of dietary nutrients. Impairment of gastric motility results in delayed gastric emptying and symptoms such as nausea, vomiting, and abdominal pain and discomfort [1]. Recent studies have shown that gastric dysmotility is one of the major pathophysiological factors in functional gastrointestinal disorders, which has a public health cost of over 30 billion dollars annually [2, 3], including functional dyspepsia [4] and gastroesophageal reflux disease [5].

Acupuncture has been practiced for thousands of years in Eastern countries and has become very popular worldwide as a complementary and alternative approach. Numerous studies on both humans and animals support the efficacy of acupuncture for treating gastrointestinal symptoms and/or diseases of gastrointestinal secretion [6, 7], sensation [8], and myoelectrical activity [9]. Acupuncture of the abdomen has been used for treating abdominal pain, suggesting that acupuncture of this site may inhibit gastric motility and/or reduce gastrospasm [10]. Recently, many studies have explored the efficacy of electroacupuncture (EA) for the treatment of gastrointestinal motility disorders and concluded that EA can alter gastrointestinal motility and improve gastrointestinal motility disorders [11]. While this therapeutic effect of EA has been proved, little is known about the underlying mechanism(s). 

The transient receptor potential vanilloid-1 (TRPV1; transient receptor potential (TRP) cation channel, subfamily V, member 1) is a member of the superfamily of TRP cation channels [12]. TRPV1 plays an important role in the modulation of EA efficacy, owing to its function in heat and mechanical sensations [13–15]. However, research on TRPV1 and EA has mainly been confined to the treatment of pain, such as cancer-related pain or hyperalgesia. Few studies have reported the relationship between EA and TRPV1 in the regulation of gastrointestinal motility. We previously reported the modulatory effects of EA on gastric motility [16]. Given the emerging role of TRPV1 receptors in mediating sensory and visceral functions, the aim of the present study was to further elucidate the mechanism of EA-mediated gastric motility modulation and to confirm whether TRPV1 was involved in this mechanism.

## 2. Materials and Methods

### 2.1. Animals

TRPV1^−/−^ mice (*n* = 30, male, 22–28 g, B6.129X1-TRPV1^tm1Jul/NJU^, J003770) and their wild-type counterparts (WT, *n* = 60, male, 22–28 g) were purchased from Model Animal Research Center of Nanjing University (Nanjing, China). Food and water were made available ad libitum, and the animals were housed under controlled environmental conditions (22°C, 40%–60% relative humidity, 12-h alternate light/dark cycles). All experimental manipulations were undertaken in accordance with the National Institutes of Health Guide for the Care and Use of Laboratory Animals and with the approval of the Scientific Investigation Board of the Nanjing University of Traditional Chinese Medicine, Nanjing, China.

### 2.2. Experimental Groups

Capsazepine injections were used in 30 of the 60 WT mice (CZP group), but not in the remaining 30 WT mice (WT group) or the 30 TRPV1 knockout mice (TRPV1^−/−^ group). Each of these three groups was divided into three subgroups of 10 mice each, and the three subgroups of each group were subjected to EA stimulation at a different intensity (1, 2, or 4 mA).

### 2.3. Experimental Procedure

The animals were fasted overnight with free access to water and anesthetized with urethane (1.2 g/kg i.p.; U2500, Sigma, USA). The trachea was cannulated to keep the respiratory tract patent, and gastric motility was recorded using a previously described method [17]. A small incision was made in the duodenum about 1-2 cm from the pylorus. A small balloon made of flexible condom rubber was inserted into the pyloric area via incision. The pressure in the balloon was measured with a transducer (YP200; Chengdu Instrument Factory, Chengdu, China) and recorded with a physiological signal-acquisition system (RM6240; Chengdu Instrument Factory) for further analysis. During the experiment, the temperature of the animal was maintained at 37°C ± 0.5°C, using an electric heating board. 

**Figure 1:**
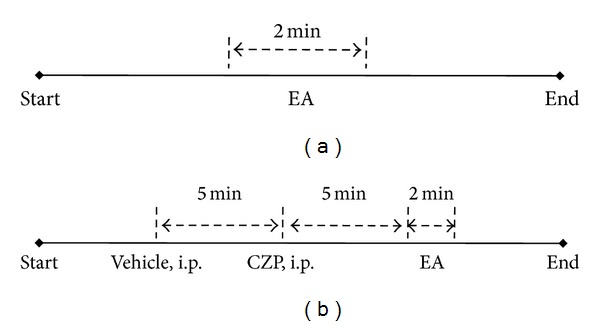
Experimental procedure. (a) Time scale of stimulation in the wild-type and TRPV1^−/−^ groups. (b) Time scale of stimulations in the capsazepine (CZP) injection group.

The experimental procedure in the WT and TRPV1^−/−^ groups is shown in Figure 1(a). The time scale of each stimulation in the capsazepine injection group is shown in Figure 1(b). Capsazepine (0.5 mg/kg, i.p.; C191, Sigma, USA) was dissolved in a vehicle (1% dimethyl sulfoxide; D8418, Sigma, USA).

### 2.4. EA Stimulation

A pair of needle electrodes (diameter, 0.3 mm) were inserted approximately 5 mm deep into the Zhongwan point (CV12), which is located at the center of the abdomen, in the midline of the body [17]. The EA intensity was set as 1, 2, or 4 mA, with alternating frequencies of 2 Hz and 15 Hz for 2 min.

### 2.5. Statistical Analysis

Data were analyzed using SPSS 17.0 software (SPSS, Chicago, USA). Differences before and after treatment were compared using a paired-sample *t*-test, and those between two groups were compared using an independent-sample *t*-test. Comparison among groups was performed using analysis of variance. *P* < 0.05 was considered statistically significant. All data were expressed as mean ± SE.

## 3. Results

### 3.1. Gastric Motility

In the resting condition (prior to EA), rhythmic gastric contractions at a frequency of 3–6/min were observed in the TRPV1^−/−^ and WT mice, and the contractile amplitude of the rhythmic waves was approximately 0.05–0.3 kPa, when balloon pressure was maintained at about 0.4–0.6 kPa by filling the balloon with 0.1–0.2 mL warm water. Injection of 0.5 mg/kg capsazepine, i.p., caused no change in gastric movement or amplitude.

### 3.2. Gastric Response to Different Intensities of EA Stimulation at CV12

**Figure 2:**
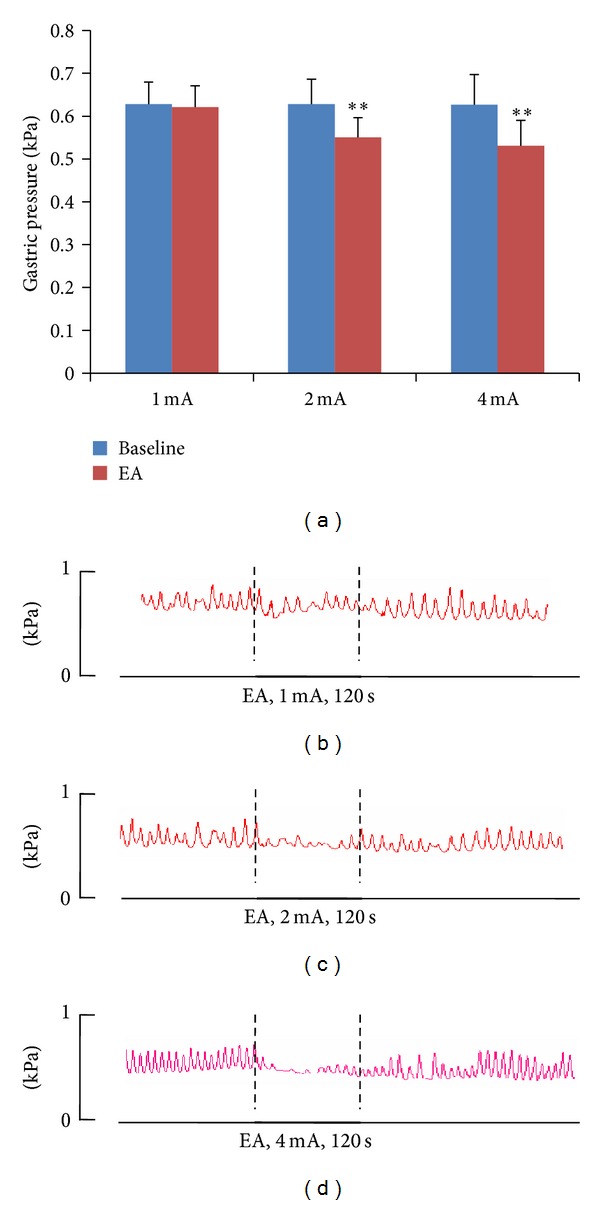
Gastric response to electroacupuncture (EA) stimulation at CV12 in wild-type mice. (a) Significant inhibition of gastric motility was induced by EA stimulation at intensities 2 mA (*n* = 10) and 4 mA (*n* = 10). ∗∗: Versus baseline, *P* < 0.001. (b)–(d) Representative examples of EA stimulation at CV12 with different intensities.

In WT mice, EA stimulation at CV12 at an intensity of 1 mA (*n* = 10) produced no significant effect on gastric motility (Figure 2(a)). In contrast, EA stimulation at 2 mA slightly inhibited gastric motility, while stimulation at 4 mA strongly inhibited it. Thus, the inhibition of gastric motility by EA stimulation was intensity dependent.

### 3.3. TRPV1 Is Involved in Gastric Motility Modulation by EA Stimulation

**Figure 3:**
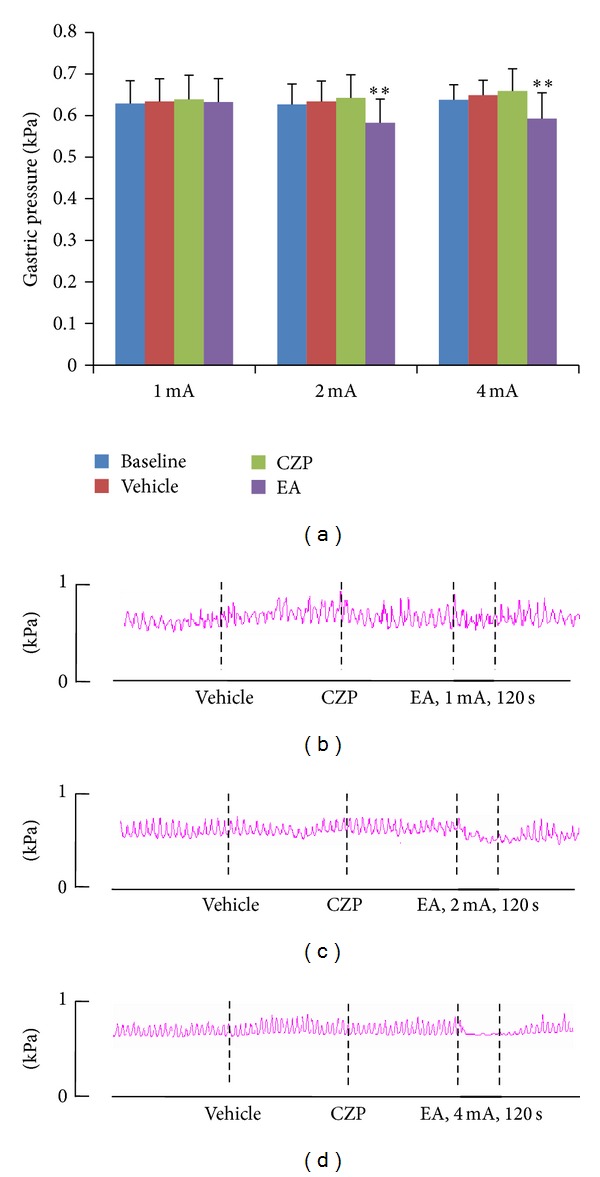
Gastric responses to electroacupuncture (EA) stimulation at CV12 after capsazepine (CZP) injection. (a) Significant inhibition of gastric motility was induced by EA at intensities 2 mA (*n* = 10) and 4 mA (*n* = 10). ∗∗: Versus CZP, *P* < 0.001. (b)–(d) Representative examples of EA stimulation at CV12 with different intensities.

**Figure 4:**
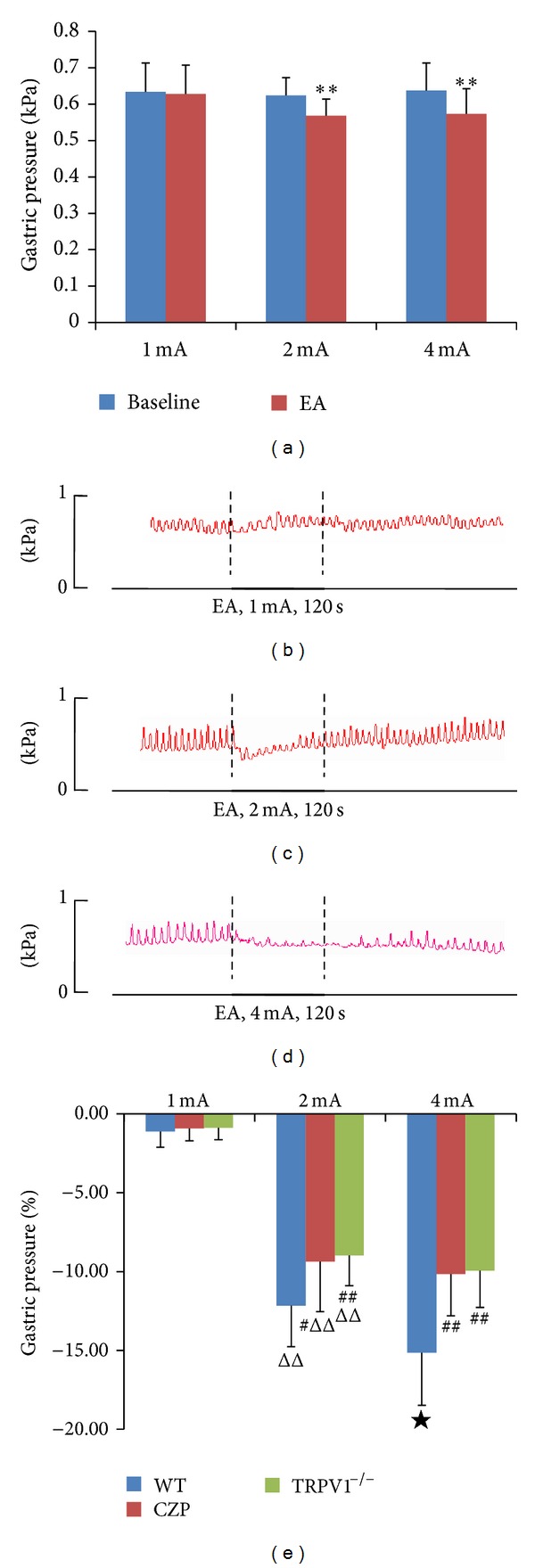
Gastric responses to electroacupuncture (EA) stimulation at CV12 in TRPV1^−/−^ mice. (a) Significant inhibition of gastric motility was induced by EA at intensities 2 mA (*n* = 10) and 4 mA (*n* = 10). ∗∗: Versus baseline, *P* < 0.001. (b)–(d) Representative examples of EA stimulation at CV12 with different intensities. (e) Percentage inhibition of gastric motility after EA stimulation at CV12 with different intensities in all three experimental groups. #: Versus WT, *P* < 0.05; ##: Versus WT, *P* < 0.01; ΔΔ: Versus 1 mA, *P* < 0.001; ★: Versus 2 mA, *P* < 0.05.

To determine the mechanism underlying the inhibitory effect of EA stimulation at CV12, we used capsazepine, a specific antagonist of TRPV1. In the WT mice, significant inhibition of gastric motility was obtained after EA stimulation at 2 and 4 mA, but not at 1 mA; moreover, this inhibitory effect was intensity dependent, being greater at 4 mA than at 2 mA (Figure 4(e)). After capsazepine injection, EA stimulation at 1 mA (*n* = 10) produced no significant inhibition, while that at 2 mA produced mild inhibition (Figure 3(a)); however, the effect of EA stimulation at 4 mA was partially blocked (Figure 4(e)). Thus, the intensity-dependent characteristic of EA stimulation disappeared after TRPV1 channel blockage. Similar results were obtained in the TRPV1^−/−^ mice (Figures 4(a)–4(e)), suggesting an important role of TRPV1 in EA-mediated modulation of gastric motility.

## 4. Discussion

The reported ameliorating effect of EA on gastric dysrhythmia has been consistent and reproducible, suggesting a robust role of EA in the treatment of gastric motility disorders [11]. The primary mechanism underlying the clinical effects of acupuncture appears to be the activation of afferent nerve fibers that innervate the skin and muscles [18]. In this study, we determined whether TRPV1 was involved in the EA-mediated regulation of gastric motility after stimulation of CV12 with different intensities. For this purpose, we used both capsazepine, which is a TRPV1 antagonist, and TRPV1 knockout mice. Acupuncture stimulation of various segmental areas of the body has been shown to alter gastric motility in anesthetized animals [19, 20]; this alteration has been facilitative or inhibitory, depending on which acupoints are stimulated. Consistent with these reports, our data showed that EA at CV12 significantly inhibited gastric motility, but only at intensities 2 and 4 mA. Moreover, this inhibitory effect was intensity dependent (Figure 4), which was in accordance with previous reports [20]. Somatic afferent nerve fibers are composed of A-*α*, A-*β*, A-*δ*, and C-fibers. The mean threshold of the action potentials of A-*δ* fibers is approximately 2 mA [20], while that of unmyelinated fibers is approximately 3 mA [21]. Consistent with this [20], EA stimulation could modulate gastric motility only at an intensity greater than the threshold for the activation of A-*δ* and/or C-fibers. In other words, the inhibitory effect of EA at 2 mA was mediated by A-*δ* fibers, while the effect of EA at 4 mA was mediated by unmyelinated fibers. 

TRPV1 mediates the transductions of intra- and extracellular signals and modulates organ functions by activating a variety of endogenous and exogenous stimuli such as mechanical stimuli, noxious heat, proteins, and capsaicin [22]. The presence of a class of visceral and somatic afferents of dorsal root origin and their functional significance in pain sensation have been well documented [23]. Recently, TRPV1 was found to be expressed at acupuncture points, indicating that it contributed to the effects of EA stimulation [24]. The above findings suggest the importance of TRPV1 in EA stimulation. To confirm whether TRPV1 was involved in the regulation of gastric motility after CV12 stimulation, we assessed the effects of EA at CV12 on gastric motility in TRPV1-null animals. After TRPV1 blockage or knockout, EA at CV12 continued to inhibit gastric motility, although, interestingly, the intensity-dependent nature of EA-mediated inhibition disappeared, especially at intensities 2 and 4 mA. Caterina et al. [25] reported that in VR1-null mice, none of the C-fibers examined were activated by capsaicin, a specific TRPV1 agonist; whereas 11 of 22 wild-type afferent nerves responded vigorously to this stimulus. Among myelinated nociceptors, only one of 13 wild-type fibers and none of the nine fibers from VR1^−/−^ mice responded to capsaicin. These results indicated that TRPV1 was mainly coexpressed with C-fibers, rather than with myelinated nociceptors [25]. Consistent with this, Koerber et al. [26] reported that TRPV1 was specifically localized in a subpopulation of C-fiber nociceptors that responded to heat (CH-fibers) but not to mechanical or cold stimuli. The majority of C-fibers innervating the skin are C-polymodal afferents that respond to both mechanical and thermal stimuli. The coexpression of TRPV1 and C-fibers combined with the identical thresholds for the elicitation of nerve action potentials and EA responses indicate that TRPV1 is involved in the intensity-dependent regulation of gastric motility by EA stimulation at CV12.
